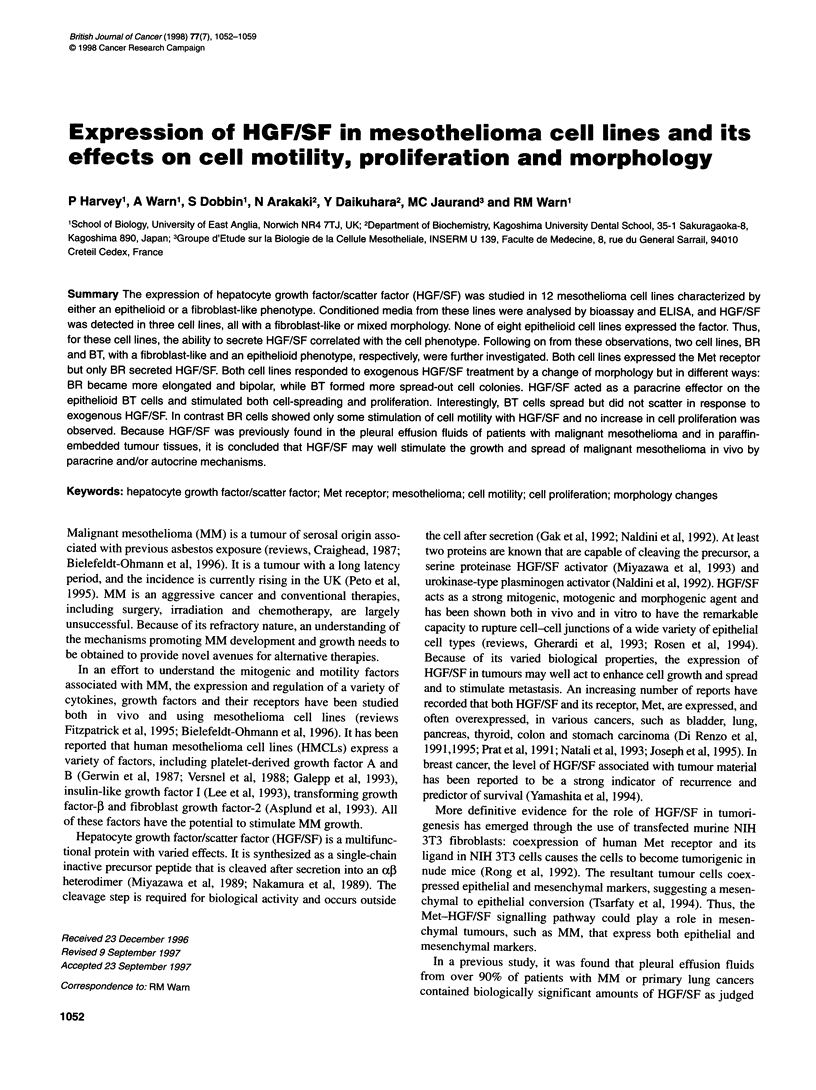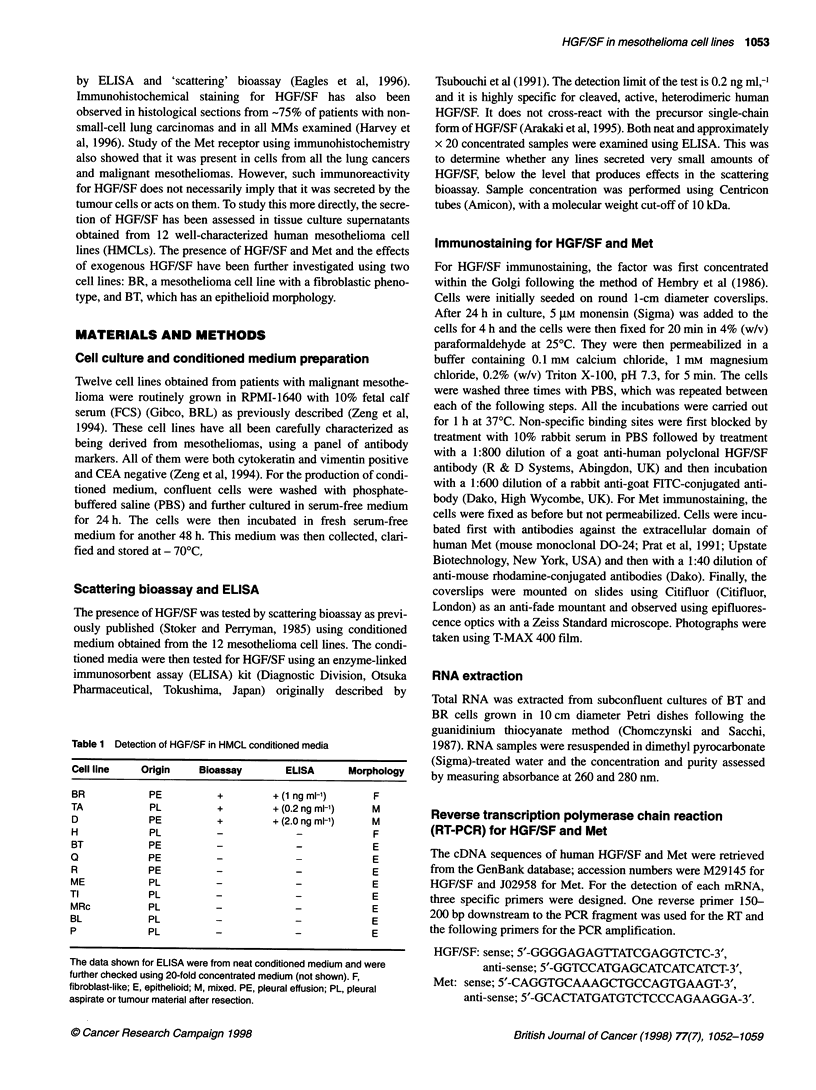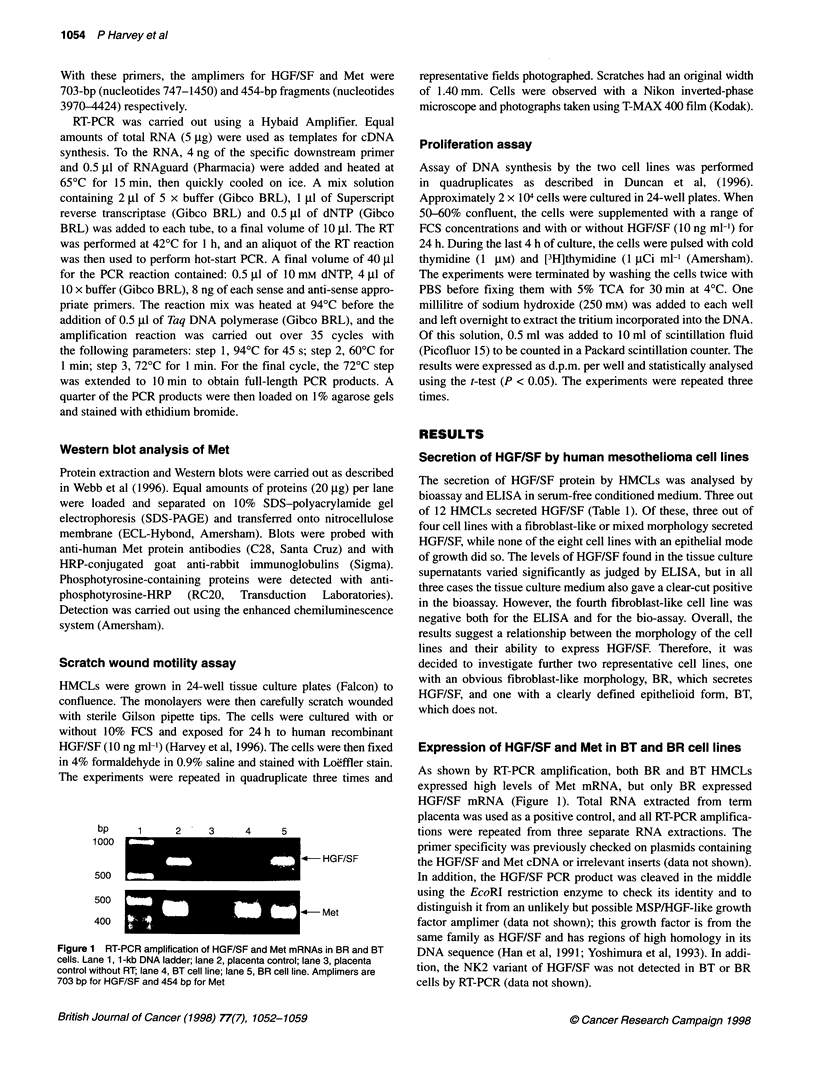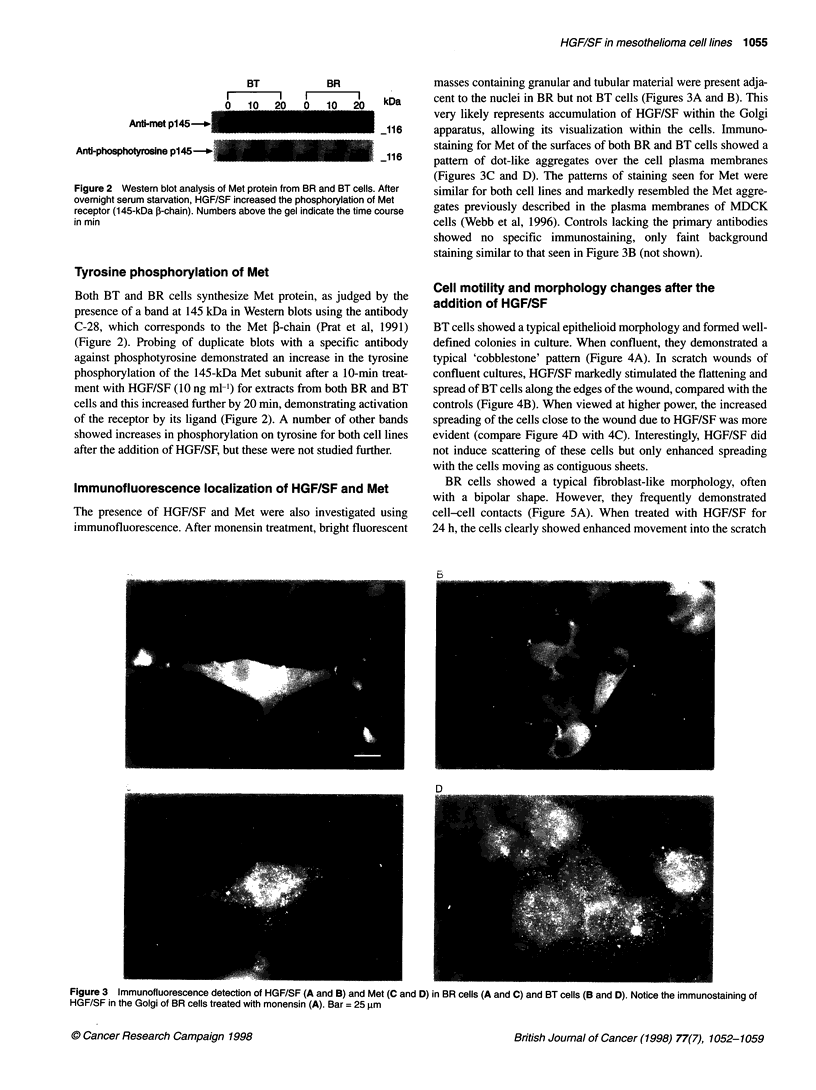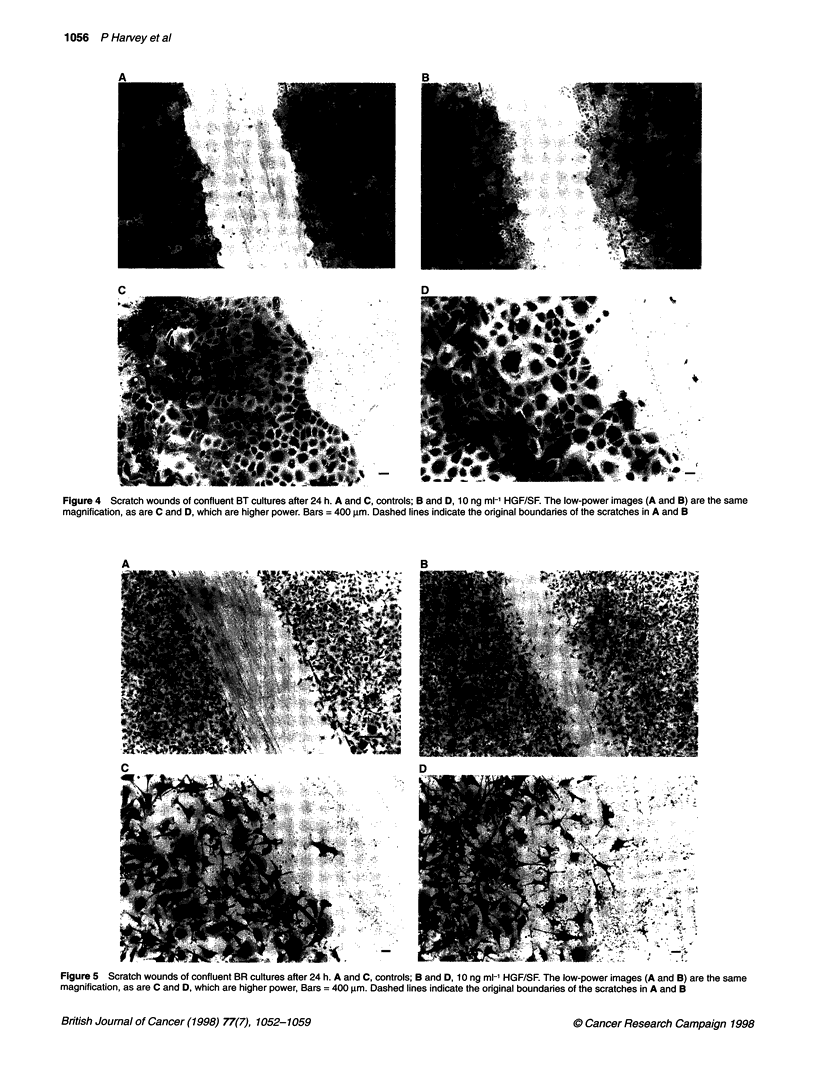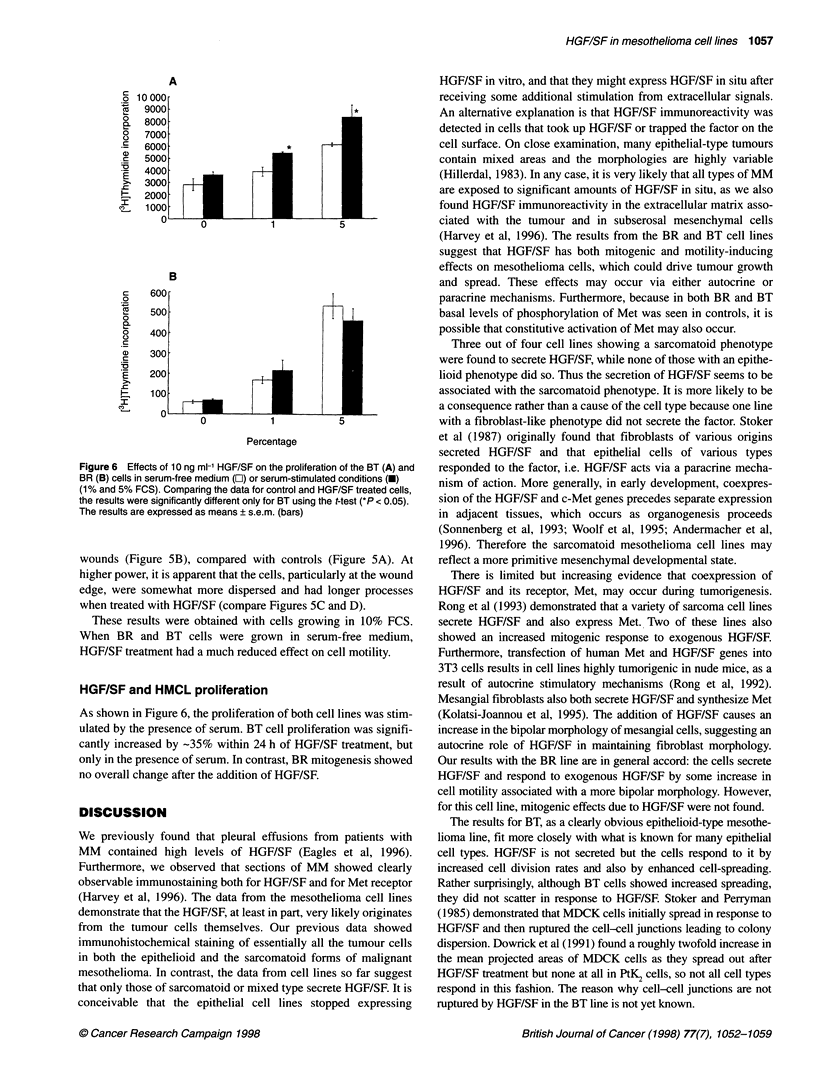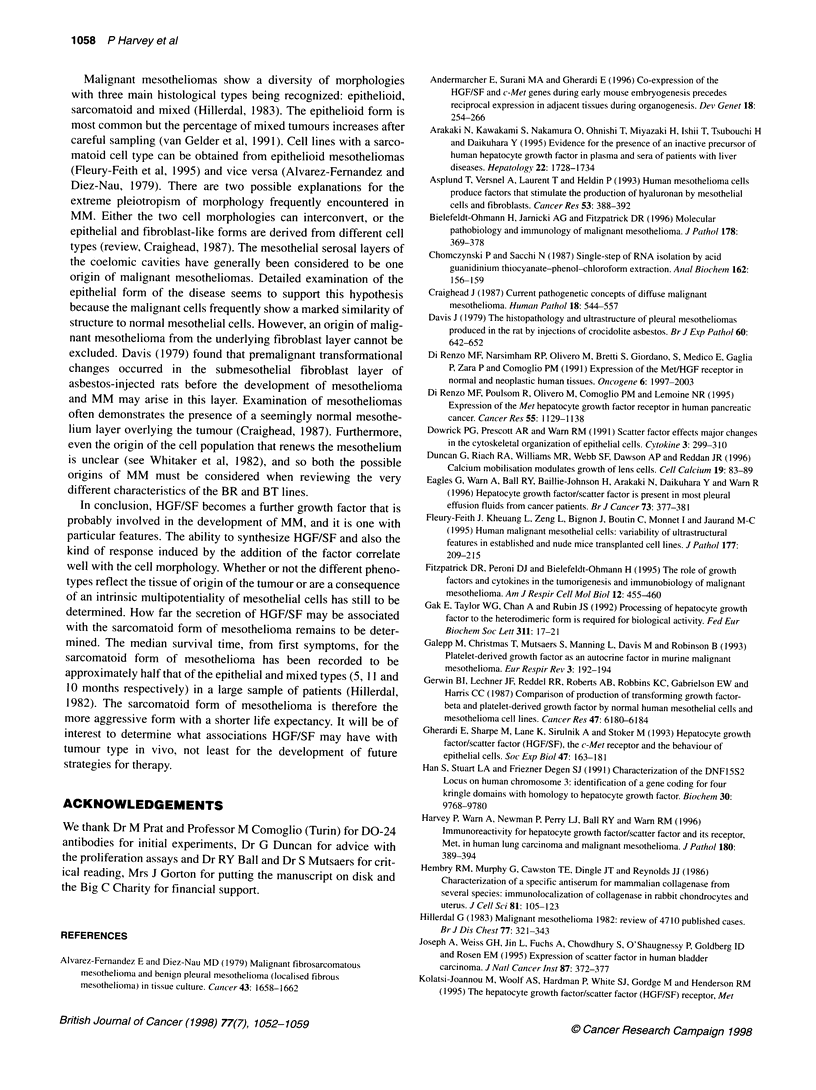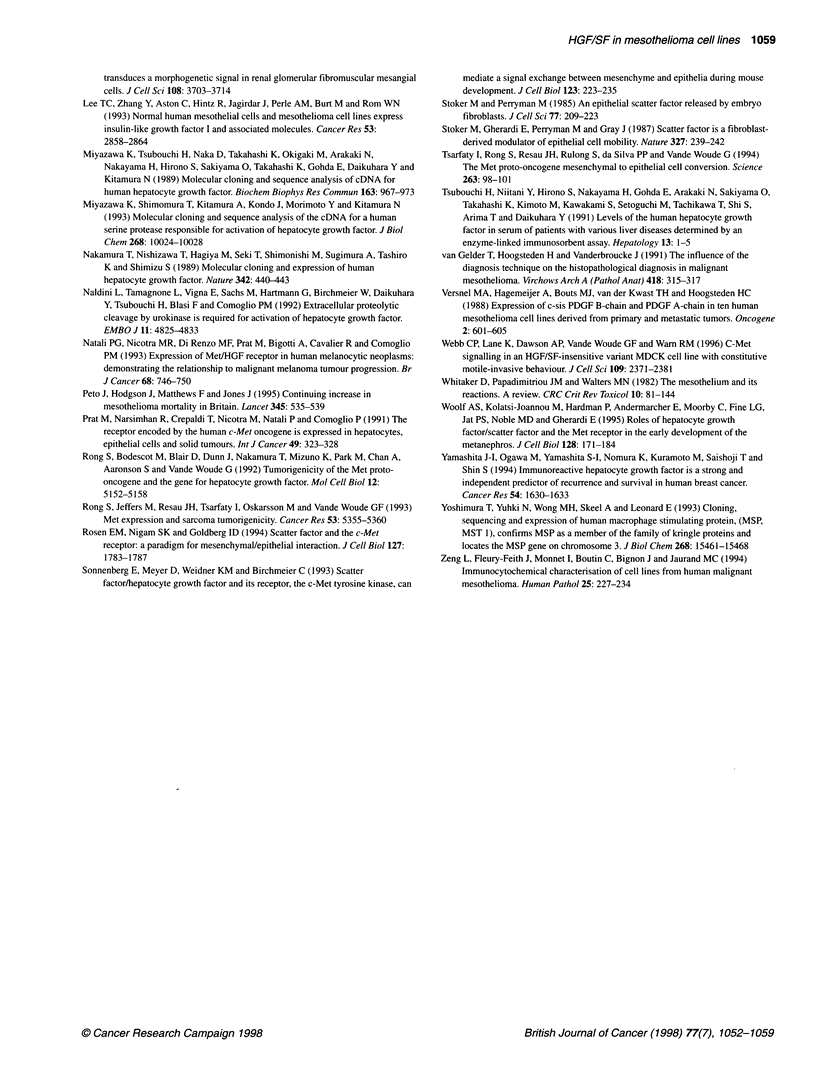# Expression of HGF/SF in mesothelioma cell lines and its effects on cell motility, proliferation and morphology.

**DOI:** 10.1038/bjc.1998.176

**Published:** 1998-04

**Authors:** P. Harvey, A. Warn, S. Dobbin, N. Arakaki, Y. Daikuhara, M. C. Jaurand, R. M. Warn

**Affiliations:** School of Biology, University of East Anglia, Norwich, UK.

## Abstract

**Images:**


British Journal of Cancer (1998) 77(7), 1052–1059
© 1998 Cancer Research Campaign

### Expression of HGF/SF in mesothelioma cell lines and its effects on cell motility, proliferation and morphology

**P Harvey[1], A Warn[1], S Dobbin[1], N Arakaki[2], Y Daikuhara[2], MC Jaurand[3] and RM Warn[1]**

[1]School of Biology, University of East Anglia, Norwich NR4 7TJ, UK; [2]Department of Biochemistry, Kagoshima University Dental School, 35-1 Sakuragaoka-8, Kagoshima 890, Japan; [3]Groupe d'Etude sur la Biologie de la Cellule Mesotheliale, INSERM U 139, Faculte de Medecine, 8, rue du General Sarrail, 94010 Creteil Cedex, France

**Summary** The expression of hepatocyte growth factor/scatter factor (HGF/SF) was studied in 12 mesothelioma cell lines characterized by either an epithelioid or a fibroblast-like phenotype. Conditioned media from these lines were analysed by bioassay and ELISA, and HGF/SF was detected in three cell lines, all with a fibroblast-like or mixed morphology. None of eight epithelioid cell lines expressed the factor. Thus, for these cell lines, the ability to secrete HGF/SF correlated with the cell phenotype. Following on from these observations, two cell lines, BR and BT, with a fibroblast-like and an epithelioid phenotype, respectively, were further investigated. Both cell lines expressed the Met receptor but only BR secreted HGF/SF. Both cell lines responded to exogenous HGF/SF treatment by a change of morphology but in different ways: BR became more elongated and bipolar, while BT formed more spread-out cell colonies. HGF/SF acted as a paracrine effector on the epithelioid BT cells and stimulated both cell-spreading and proliferation. Interestingly, BT cells spread but did not scatter in response to exogenous HGF/SF. In contrast BR cells showed only some stimulation of cell motility with HGF/SF and no increase in cell proliferation was observed. Because HGF/SF was previously found in the pleural effusion fluids of patients with malignant mesothelioma and in paraffin-embedded tumour tissues, it is concluded that HGF/SF may well stimulate the growth and spread of malignant mesothelioma in vivo by paracrine and/or autocrine mechanisms.

**Keywords:** hepatocyte growth factor/scatter factor; Met receptor; mesothelioma; cell motility; cell proliferation; morphology changes

Malignant mesothelioma (MM) is a tumour of serosal origin associated with previous asbestos exposure (reviews, Craighead, 1987; Bielefeldt-Ohmann et al, 1996). It is a tumour with a long latency period, and the incidence is currently rising in the UK (Peto et al, 1995). MM is an aggressive cancer and conventional therapies, including surgery, irradiation and chemotherapy, are largely unsuccessful. Because of its refractory nature, an understanding of the mechanisms promoting MM development and growth needs to be obtained to provide novel avenues for alternative therapies.

In an effort to understand the mitogenic and motility factors associated with MM, the expression and regulation of a variety of cytokines, growth factors and their receptors have been studied both in vivo and using mesothelioma cell lines (reviews Fitzpatrick et al, 1995; Bielefeldt-Ohmann et al, 1996). It has been reported that human mesothelioma cell lines (HMCLs) express a variety of factors, including platelet-derived growth factor A and B (Gerwin et al, 1987; Versnel et al, 1988; Galepp et al, 1993), insulin-like growth factor I (Lee et al, 1993), transforming growth factor-$\beta$ and fibroblast growth factor-2 (Asplund et al, 1993). All of these factors have the potential to stimulate MM growth.

Hepatocyte growth factor/scatter factor (HGF/SF) is a multifunctional protein with varied effects. It is synthesized as a single-chain inactive precursor peptide that is cleaved after secretion into an $\alpha\beta$ heterodimer (Miyazawa et al, 1989; Nakamura et al, 1989). The cleavage step is required for biological activity and occurs outside the cell after secretion (Gak et al, 1992; Naldini et al, 1992). At least two proteins are known that are capable of cleaving the precursor, a serine proteinase HGF/SF activator (Miyazawa et al, 1993) and urokinase-type plasminogen activator (Naldini et al, 1992). HGF/SF acts as a strong mitogenic, motogenic and morphogenic agent and has been shown both in vivo and in vitro to have the remarkable capacity to rupture cell–cell junctions of a wide variety of epithelial cell types (reviews, Gherardi et al, 1993; Rosen et al, 1994). Because of its varied biological properties, the expression of HGF/SF in tumours may well act to enhance cell growth and spread and to stimulate metastasis. An increasing number of reports have recorded that both HGF/SF and its receptor, Met, are expressed, and often overexpressed, in various cancers, such as bladder, lung, pancreas, thyroid, colon and stomach carcinoma (Di Renzo et al, 1991,1995; Prat et al, 1991; Natali et al, 1993; Joseph et al, 1995). In breast cancer, the level of HGF/SF associated with tumour material has been reported to be a strong indicator of recurrence and predictor of survival (Yamashita et al, 1994).

More definitive evidence for the role of HGF/SF in tumorigenesis has emerged through the use of transfected murine NIH 3T3 fibroblasts: coexpression of human Met receptor and its ligand in NIH 3T3 cells causes the cells to become tumorigenic in nude mice (Rong et al, 1992). The resultant tumour cells coexpressed epithelial and mesenchymal markers, suggesting a mesenchymal to epithelial conversion (Tsarfaty et al, 1994). Thus, the Met–HGF/SF signalling pathway could play a role in mesenchymal tumours, such as MM, that express both epithelial and mesenchymal markers.

In a previous study, it was found that pleural effusion fluids from over 90% of patients with MM or primary lung cancers contained biologically significant amounts of HGF/SF as judged

Received 23 December 1996
Revised 9 September 1997
Accepted 23 September 1997

Correspondence to: RM Warn by ELISA and 'scattering' bioassay (Eagles et al, 1996). Immunohistochemical staining for HGF/SF has also been observed in histological sections from ~75% of patients with non-small-cell lung carcinomas and in all MMs examined (Harvey et al, 1996). Study of the Met receptor using immunohistochemistry also showed that it was present in cells from all the lung cancers and malignant mesotheliomas. However, such immunoreactivity for HGF/SF does not necessarily imply that it was secreted by the tumour cells or acts on them. To study this more directly, the secretion of HGF/SF has been assessed in tissue culture supernatants obtained from 12 well-characterized human mesothelioma cell lines (HMCLs). The presence of HGF/SF and Met and the effects of exogenous HGF/SF have been further investigated using two cell lines: BR, a mesothelioma cell line with a fibroblastic phenotype, and BT, which has an epithelioid morphology.

### MATERIALS AND METHODS

### Cell culture and conditioned medium preparation

Twelve cell lines obtained from patients with malignant mesothelioma were routinely grown in RPMI-1640 with 10% fetal calf serum (FCS) (Gibco, BRL) as previously described (Zeng et al, 1994). These cell lines have all been carefully characterized as being derived from mesotheliomas, using a panel of antibody markers. All of them were both cytokeratin and vimentin positive and CEA negative (Zeng et al, 1994). For the production of conditioned medium, confluent cells were washed with phosphate-buffered saline (PBS) and further cultured in serum-free medium for 24 h. The cells were then incubated in fresh serum-free medium for another 48 h. This medium was then collected, clarified and stored at – 70°C.

### Scattering bioassay and ELISA

The presence of HGF/SF was tested by scattering bioassay as previously published (Stoker and Perryman, 1985) using conditioned medium obtained from the 12 mesothelioma cell lines. The conditioned media were then tested for HGF/SF using an enzyme-linked immunosorbent assay (ELISA) kit (Diagnostic Division, Otsuka Pharmaceutical, Tokushima, Japan) originally described by Tsubouchi et al (1991). The detection limit of the test is 0.2 ng ml,$^{-1}$ and it is highly specific for cleaved, active, heterodimeric human HGF/SF. It does not cross-react with the precursor single-chain form of HGF/SF (Arakaki et al, 1995). Both neat and approximately × 20 concentrated samples were examined using ELISA. This was to determine whether any lines secreted very small amounts of HGF/SF, below the level that produces effects in the scattering bioassay. Sample concentration was performed using Centricon tubes (Amicon), with a molecular weight cut-off of 10 kDa.

**Table 1** Detection of HGF/SF in HMCL conditioned media

| Cell line | Origin | Bioassay | ELISA | Morphology |
|---|---|---|---|---|
| BR | PE | + | + (1 ng ml$^{-1}$) | F |
| TA | PL | + | + (0.2 ng ml$^{-1}$) | M |
| D | PE | + | + (2.0 ng ml$^{-1}$) | M |
| H | PL | – | – | F |
| BT | PE | – | – | E |
| Q | PE | – | – | E |
| R | PE | – | – | E |
| ME | PL | – | – | E |
| TI | PL | – | – | E |
| MRc | PL | – | – | E |
| BL | PL | – | – | E |
| P | PL | – | – | E |

The data shown for ELISA were from neat conditioned medium and were further checked using 20-fold concentrated medium (not shown). F, fibroblast-like; E, epithelioid; M, mixed. PE, pleural effusion; PL, pleural aspirate or tumour material after resection.

### Immunostaining for HGF/SF and Met

For HGF/SF immunostaining, the factor was first concentrated within the Golgi following the method of Hembry et al (1986). Cells were initially seeded on round 1-cm diameter coverslips. After 24 h in culture, 5 μM monensin (Sigma) was added to the cells for 4 h and the cells were then fixed for 20 min in 4% (w/v) paraformaldehyde at 25°C. They were then permeabilized in a buffer containing 0.1 mM calcium chloride, 1 mM magnesium chloride, 0.2% (w/v) Triton X-100, pH 7.3, for 5 min. The cells were washed three times with PBS, which was repeated between each of the following steps. All the incubations were carried out for 1 h at 37°C. Non-specific binding sites were first blocked by treatment with 10% rabbit serum in PBS followed by treatment with a 1:800 dilution of a goat anti-human polyclonal HGF/SF antibody (R & D Systems, Abingdon, UK) and then incubation with a 1:600 dilution of a rabbit anti-goat FITC-conjugated antibody (Dako, High Wycombe, UK). For Met immunostaining, the cells were fixed as before but not permeabilized. Cells were incubated first with antibodies against the extracellular domain of human Met (mouse monoclonal DO-24; Prat et al, 1991; Upstate Biotechnology, New York, USA) and then with a 1:40 dilution of anti-mouse rhodamine-conjugated antibodies (Dako). Finally, the coverslips were mounted on slides using Citifluor (Citifluor, London) as an anti-fade mountant and observed using epifluorescence optics with a Zeiss Standard microscope. Photographs were taken using T-MAX 400 film.

### RNA extraction

Total RNA was extracted from subconfluent cultures of BT and BR cells grown in 10 cm diameter Petri dishes following the guanidinium thiocyanate method (Chomczynski and Sacchi, 1987). RNA samples were resuspended in dimethyl pyrocarbonate (Sigma)-treated water and the concentration and purity assessed by measuring absorbance at 260 and 280 nm.

### Reverse transcription polymerase chain reaction (RT-PCR) for HGF/SF and Met

The cDNA sequences of human HGF/SF and Met were retrieved from the GenBank database; accession numbers were M29145 for HGF/SF and J02958 for Met. For the detection of each mRNA, three specific primers were designed. One reverse primer 150–200 bp downstream to the PCR fragment was used for the RT and the following primers for the PCR amplification.

HGF/SF: sense; 5′-GGGGAGAGTTATCGAGGTCTC-3′,
anti-sense; 5′-GGTCCATGAGCATCATCATCT-3′,
Met: sense; 5′-CAGGTGCAAAGCTGCCAGTGAAGT-3′,
anti-sense; 5′-GCACTATGATGTCTCCCAGAAGGA-3′.

© Cancer Research Campaign 1998

With these primers, the amplimers for HGF/SF and Met were 703-bp (nucleotides 747–1450) and 454-bp fragments (nucleotides 3970–4424) respectively.

RT-PCR was carried out using a Hybaid Amplifier. Equal amounts of total RNA (5 µg) were used as templates for cDNA synthesis. To the RNA, 4 ng of the specific downstream primer and 0.5 µl of RNAguard (Pharmacia) were added and heated at 65°C for 15 min, then quickly cooled on ice. A mix solution containing 2 µl of 5 × buffer (Gibco BRL), 1 µl of Superscript reverse transcriptase (Gibco BRL) and 0.5 µl of dNTP (Gibco BRL) was added to each tube, to a final volume of 10 µl. The RT was performed at 42°C for 1 h, and an aliquot of the RT reaction was then used to perform hot-start PCR. A final volume of 40 µl for the PCR reaction contained: 0.5 µl of 10 mM dNTP, 4 µl of 10 × buffer (Gibco BRL), 8 ng of each sense and anti-sense appropriate primers. The reaction mix was heated at 94°C before the addition of 0.5 µl of *Taq* DNA polymerase (Gibco BRL), and the amplification reaction was carried out over 35 cycles with the following parameters: step 1, 94°C for 45 s; step 2, 60°C for 1 min; step 3, 72°C for 1 min. For the final cycle, the 72°C step was extended to 10 min to obtain full-length PCR products. A quarter of the PCR products were then loaded on 1% agarose gels and stained with ethidium bromide.

### Western blot analysis of Met

Protein extraction and Western blots were carried out as described in Webb et al (1996). Equal amounts of proteins (20 µg) per lane were loaded and separated on 10% SDS–polyacrylamide gel electrophoresis (SDS-PAGE) and transferred onto nitrocellulose membrane (ECL-Hybond, Amersham). Blots were probed with anti-human Met protein antibodies (C28, Santa Cruz) and with HRP-conjugated goat anti-rabbit immunoglobulins (Sigma). Phosphotyrosine-containing proteins were detected with anti-phosphotyrosine-HRP (RC20, Transduction Laboratories). Detection was carried out using the enhanced chemiluminescence system (Amersham).

### Scratch wound motility assay

HMCLs were grown in 24-well tissue culture plates (Falcon) to confluence. The monolayers were then carefully scratch wounded with sterile Gilson pipette tips. The cells were cultured with or without 10% FCS and exposed for 24 h to human recombinant HGF/SF (10 ng ml$^{-1}$) (Harvey et al, 1996). The cells were then fixed in 4% formaldehyde in 0.9% saline and stained with Loëffler stain. The experiments were repeated in quadruplicate three times and representative fields photographed. Scratches had an original width of 1.40 mm. Cells were observed with a Nikon inverted-phase microscope and photographs taken using T-MAX 400 film (Kodak).

**Figure 1** RT-PCR amplification of HGF/SF and Met mRNAs in BR and BT cells. Lane 1, 1-kb DNA ladder; lane 2, placenta control; lane 3, placenta control without RT; lane 4, BT cell line; lane 5, BR cell line. Amplimers are 703 bp for HGF/SF and 454 bp for Met

### Proliferation assay

Assay of DNA synthesis by the two cell lines was performed in quadruplicates as described in Duncan et al, (1996). Approximately 2 × 10$^4$ cells were cultured in 24-well plates. When 50–60% confluent, the cells were supplemented with a range of FCS concentrations and with or without HGF/SF (10 ng ml$^{-1}$) for 24 h. During the last 4 h of culture, the cells were pulsed with cold thymidine (1 µM) and [$^3$H]thymidine (1 µCi ml$^{-1}$ (Amersham). The experiments were terminated by washing the cells twice with PBS before fixing them with 5% TCA for 30 min at 4°C. One millilitre of sodium hydroxide (250 mM) was added to each well and left overnight to extract the tritium incorporated into the DNA. Of this solution, 0.5 ml was added to 10 ml of scintillation fluid (Picofluor 15) to be counted in a Packard scintillation counter. The results were expressed as d.p.m. per well and statistically analysed using the *t*-test ($P < 0.05$). The experiments were repeated three times.

### RESULTS

### Secretion of HGF/SF by human mesothelioma cell lines

The secretion of HGF/SF protein by HMCLs was analysed by bioassay and ELISA in serum-free conditioned medium. Three out of 12 HMCLs secreted HGF/SF (Table 1). Of these, three out of four cell lines with a fibroblast-like or mixed morphology secreted HGF/SF, while none of the eight cell lines with an epithelial mode of growth did so. The levels of HGF/SF found in the tissue culture supernatants varied significantly as judged by ELISA, but in all three cases the tissue culture medium also gave a clear-cut positive in the bioassay. However, the fourth fibroblast-like cell line was negative both for the ELISA and for the bio-assay. Overall, the results suggest a relationship between the morphology of the cell lines and their ability to express HGF/SF. Therefore, it was decided to investigate further two representative cell lines, one with an obvious fibroblast-like morphology, BR, which secretes HGF/SF, and one with a clearly defined epithelioid form, BT, which does not.

### Expression of HGF/SF and Met in BT and BR cell lines

As shown by RT-PCR amplification, both BR and BT HMCLs expressed high levels of Met mRNA, but only BR expressed HGF/SF mRNA (Figure 1). Total RNA extracted from term placenta was used as a positive control, and all RT-PCR amplifications were repeated from three separate RNA extractions. The primer specificity was previously checked on plasmids containing the HGF/SF and Met cDNA or irrelevant inserts (data not shown). In addition, the HGF/SF PCR product was cleaved in the middle using the *Eco*RI restriction enzyme to check its identity and to distinguish it from an unlikely but possible MSP/HGF-like growth factor amplimer (data not shown); this growth factor is from the same family as HGF/SF and has regions of high homology in its DNA sequence (Han et al, 1991; Yoshimura et al, 1993). In addition, the NK2 variant of HGF/SF was not detected in BT or BR cells by RT-PCR (data not shown).

© Cancer Research Campaign 1998

**Figure 2** Western blot analysis of Met protein from BR and BT cells. After overnight serum starvation, HGF/SF increased the phosphorylation of Met receptor (145-kDa β-chain). Numbers above the gel indicate the time course in min

### Tyrosine phosphorylation of Met

Both BT and BR cells synthesize Met protein, as judged by the presence of a band at 145 kDa in Western blots using the antibody C-28, which corresponds to the Met β-chain (Prat et al, 1991) (Figure 2). Probing of duplicate blots with a specific antibody against phosphotyrosine demonstrated an increase in the tyrosine phosphorylation of the 145-kDa Met subunit after a 10-min treatment with HGF/SF (10 ng $ml^{-1}$) for extracts from both BR and BT cells and this increased further by 20 min, demonstrating activation of the receptor by its ligand (Figure 2). A number of other bands showed increases in phosphorylation on tyrosine for both cell lines after the addition of HGF/SF, but these were not studied further.

### Immunofluorescence localization of HGF/SF and Met

The presence of HGF/SF and Met were also investigated using immunofluorescence. After monensin treatment, bright fluorescent masses containing granular and tubular material were present adjacent to the nuclei in BR but not BT cells (Figures 3A and B). This very likely represents accumulation of HGF/SF within the Golgi apparatus, allowing its visualization within the cells. Immunostaining for Met of the surfaces of both BR and BT cells showed a pattern of dot-like aggregates over the cell plasma membranes (Figures 3C and D). The patterns of staining seen for Met were similar for both cell lines and markedly resembled the Met aggregates previously described in the plasma membranes of MDCK cells (Webb et al, 1996). Controls lacking the primary antibodies showed no specific immunostaining, only faint background staining similar to that seen in Figure 3B (not shown).

### Cell motility and morphology changes after the addition of HGF/SF

BT cells showed a typical epithelioid morphology and formed well-defined colonies in culture. When confluent, they demonstrated a typical 'cobblestone' pattern (Figure 4A). In scratch wounds of confluent cultures, HGF/SF markedly stimulated the flattening and spread of BT cells along the edges of the wound, compared with the controls (Figure 4B). When viewed at higher power, the increased spreading of the cells close to the wound due to HGF/SF was more evident (compare Figure 4D with 4C). Interestingly, HGF/SF did not induce scattering of these cells but only enhanced spreading with the cells moving as contiguous sheets.

BR cells showed a typical fibroblast-like morphology, often with a bipolar shape. However, they frequently demonstrated cell–cell contacts (Figure 5A). When treated with HGF/SF for 24 h, the cells clearly showed enhanced movement into the scratch

**Figure 3** Immunofluorescence detection of HGF/SF (**A** and **B**) and Met (**C** and **D**) in BR cells (**A** and **C**) and BT cells (**B** and **D**). Notice the immunostaining of HGF/SF in the Golgi of BR cells treated with monensin (**A**). Bar = 25 μm

**Figure 4** Scratch wounds of confluent BT cultures after 24 h. **A** and **C**, controls; **B** and **D**, 10 ng ml$^{-1}$ HGF/SF. The low-power images (**A** and **B**) are the same magnification, as are **C** and **D**, which are higher power. Bars = 400 μm. Dashed lines indicate the original boundaries of the scratches in **A** and **B**

**Figure 5** Scratch wounds of confluent BR cultures after 24 h. **A** and **C**, controls; **B** and **D**, 10 ng ml$^{-1}$ HGF/SF. The low-power images (**A** and **B**) are the same magnification, as are **C** and **D**, which are higher power, Bars = 400 μm. Dashed lines indicate the original boundaries of the scratches in **A** and **B**

© Cancer Research Campaign 1998

**Figure 6** Effects of 10 ng ml$^{-1}$ HGF/SF on the proliferation of the BT (**A**) and BR (**B**) cells in serum-free medium (□) or serum-stimulated conditions (■) (1% and 5% FCS). Comparing the data for control and HGF/SF treated cells, the results were significantly different only for BT using the *t*-test (*$P < 0.05$). The results are expressed as means ± s.e.m. (bars)

wounds (Figure 5B), compared with controls (Figure 5A). At higher power, it is apparent that the cells, particularly at the wound edge, were somewhat more dispersed and had longer processes when treated with HGF/SF (compare Figures 5C and D).

These results were obtained with cells growing in 10% FCS. When BR and BT cells were grown in serum-free medium, HGF/SF treatment had a much reduced effect on cell motility.

### HGF/SF and HMCL proliferation

As shown in Figure 6, the proliferation of both cell lines was stimulated by the presence of serum. BT cell proliferation was significantly increased by ~35% within 24 h of HGF/SF treatment, but only in the presence of serum. In contrast, BR mitogenesis showed no overall change after the addition of HGF/SF.

### DISCUSSION

We previously found that pleural effusions from patients with MM contained high levels of HGF/SF (Eagles et al, 1996). Furthermore, we observed that sections of MM showed clearly observable immunostaining both for HGF/SF and for Met receptor (Harvey et al, 1996). The data from the mesothelioma cell lines demonstrate that the HGF/SF, at least in part, very likely originates from the tumour cells themselves. Our previous data showed immunohistochemical staining of essentially all the tumour cells in both the epithelioid and the sarcomatoid forms of malignant mesothelioma. In contrast, the data from cell lines so far suggest that only those of sarcomatoid or mixed type secrete HGF/SF. It is conceivable that the epithelial cell lines stopped expressing HGF/SF in vitro, and that they might express HGF/SF in situ after receiving some additional stimulation from extracellular signals. An alternative explanation is that HGF/SF immunoreactivity was detected in cells that took up HGF/SF or trapped the factor on the cell surface. On close examination, many epithelial-type tumours contain mixed areas and the morphologies are highly variable (Hillerdal, 1983). In any case, it is very likely that all types of MM are exposed to significant amounts of HGF/SF in situ, as we also found HGF/SF immunoreactivity in the extracellular matrix associated with the tumour and in subserosal mesenchymal cells (Harvey et al, 1996). The results from the BR and BT cell lines suggest that HGF/SF has both mitogenic and motility-inducing effects on mesothelioma cells, which could drive tumour growth and spread. These effects may occur via either autocrine or paracrine mechanisms. Furthermore, because in both BR and BT basal levels of phosphorylation of Met was seen in controls, it is possible that constitutive activation of Met may also occur.

Three out of four cell lines showing a sarcomatoid phenotype were found to secrete HGF/SF, while none of those with an epithelioid phenotype did so. Thus the secretion of HGF/SF seems to be associated with the sarcomatoid phenotype. It is more likely to be a consequence rather than a cause of the cell type because one line with a fibroblast-like phenotype did not secrete the factor. Stoker et al (1987) originally found that fibroblasts of various origins secreted HGF/SF and that epithelial cells of various types responded to the factor, i.e. HGF/SF acts via a paracrine mechanism of action. More generally, in early development, coexpression of the HGF/SF and c-Met genes precedes separate expression in adjacent tissues, which occurs as organogenesis proceeds (Sonnenberg et al, 1993; Woolf et al, 1995; Andermacher et al, 1996). Therefore the sarcomatoid mesothelioma cell lines may reflect a more primitive mesenchymal developmental state.

There is limited but increasing evidence that coexpression of HGF/SF and its receptor, Met, may occur during tumorigenesis. Rong et al (1993) demonstrated that a variety of sarcoma cell lines secrete HGF/SF and also express Met. Two of these lines also showed an increased mitogenic response to exogenous HGF/SF. Furthermore, transfection of human Met and HGF/SF genes into 3T3 cells results in cell lines highly tumorigenic in nude mice, as a result of autocrine stimulatory mechanisms (Rong et al, 1992). Mesangial fibroblasts also both secrete HGF/SF and synthesize Met (Kolatsi-Joannou et al, 1995). The addition of HGF/SF causes an increase in the bipolar morphology of mesangial cells, suggesting an autocrine role of HGF/SF in maintaining fibroblast morphology. Our results with the BR line are in general accord: the cells secrete HGF/SF and respond to exogenous HGF/SF by some increase in cell motility associated with a more bipolar morphology. However, for this cell line, mitogenic effects due to HGF/SF were not found.

The results for BT, as a clearly obvious epithelioid-type mesothelioma line, fit more closely with what is known for many epithelial cell types. HGF/SF is not secreted but the cells respond to it by increased cell division rates and also by enhanced cell-spreading. Rather surprisingly, although BT cells showed increased spreading, they did not scatter in response to HGF/SF. Stoker and Perryman (1985) demonstrated that MDCK cells initially spread in response to HGF/SF and then ruptured the cell–cell junctions leading to colony dispersion. Dowrick et al (1991) found a roughly twofold increase in the mean projected areas of MDCK cells as they spread out after HGF/SF treatment but none at all in $PtK_2$ cells, so not all cell types respond in this fashion. The reason why cell–cell junctions are not ruptured by HGF/SF in the BT line is not yet known.

Malignant mesotheliomas show a diversity of morphologies with three main histological types being recognized: epithelioid, sarcomatoid and mixed (Hillerdal, 1983). The epithelioid form is most common but the percentage of mixed tumours increases after careful sampling (van Gelder et al, 1991). Cell lines with a sarcomatoid cell type can be obtained from epithelioid mesotheliomas (Fleury-Feith et al, 1995) and vice versa (Alvarez-Fernandez and Diez-Nau, 1979). There are two possible explanations for the extreme pleiotropism of morphology frequently encountered in MM. Either the two cell morphologies can interconvert, or the epithelial and fibroblast-like forms are derived from different cell types (review, Craighead, 1987). The mesothelial serosal layers of the coelomic cavities have generally been considered to be one origin of malignant mesotheliomas. Detailed examination of the epithelial form of the disease seems to support this hypothesis because the malignant cells frequently show a marked similarity of structure to normal mesothelial cells. However, an origin of malignant mesothelioma from the underlying fibroblast layer cannot be excluded. Davis (1979) found that premalignant transformational changes occurred in the submesothelial fibroblast layer of asbestos-injected rats before the development of mesothelioma and MM may arise in this layer. Examination of mesotheliomas often demonstrates the presence of a seemingly normal mesothelium layer overlying the tumour (Craighead, 1987). Furthermore, even the origin of the cell population that renews the mesothelium is unclear (see Whitaker et al, 1982), and so both the possible origins of MM must be considered when reviewing the very different characteristics of the BR and BT lines.

In conclusion, HGF/SF becomes a further growth factor that is probably involved in the development of MM, and it is one with particular features. The ability to synthesize HGF/SF and also the kind of response induced by the addition of the factor correlate well with the cell morphology. Whether or not the different phenotypes reflect the tissue of origin of the tumour or are a consequence of an intrinsic multipotentiality of mesothelial cells has still to be determined. How far the secretion of HGF/SF may be associated with the sarcomatoid form of mesothelioma remains to be determined. The median survival time, from first symptoms, for the sarcomatoid form of mesothelioma has been recorded to be approximately half that of the epithelial and mixed types (5, 11 and 10 months respectively) in a large sample of patients (Hillerdal, 1982). The sarcomatoid form of mesothelioma is therefore the more aggressive form with a shorter life expectancy. It will be of interest to determine what associations HGF/SF may have with tumour type in vivo, not least for the development of future strategies for therapy.

### ACKNOWLEDGEMENTS

We thank Dr M Prat and Professor M Comoglio (Turin) for DO-24 antibodies for initial experiments, Dr G Duncan for advice with the proliferation assays and Dr RY Ball and Dr S Mutsaers for critical reading, Mrs J Gorton for putting the manuscript on disk and the Big C Charity for financial support.

### REFERENCES

Alvarez-Fernandez E and Diez-Nau MD (1979) Malignant fibrosarcomatous mesothelioma and benign pleural mesothelioma (localised fibrous mesothelioma) in tissue culture. *Cancer* **43**: 1658–1662

Andermarcher E, Surani MA and Gherardi E (1996) Co-expression of the HGF/SF and *c-Met* genes during early mouse embryogenesis precedes reciprocal expression in adjacent tissues during organogenesis. *Dev Genet* **18**: 254–266

Arakaki N, Kawakami S, Nakamura O, Ohnishi T, Miyazaki H, Ishii T, Tsubouchi H and Daikuhara Y (1995) Evidence for the presence of an inactive precursor of human hepatocyte growth factor in plasma and sera of patients with liver diseases. *Hepatology* **22**: 1728–1734

Asplund T, Versnel A, Laurent T and Heldin P (1993) Human mesothelioma cells produce factors that stimulate the production of hyaluronan by mesothelial cells and fibroblasts. *Cancer Res* **53**: 388–392

Bielefeldt-Ohmann H, Jarnicki AG and Fitzpatrick DR (1996) Molecular pathobiology and immunology of malignant mesothelioma. *J Pathol* **178**: 369–378

Chomczynski P and Sacchi N (1987) Single-step of RNA isolation by acid guanidinium thiocyanate–phenol–chloroform extraction. *Anal Biochem* **162**: 156–159

Craighead J (1987) Current pathogenetic concepts of diffuse malignant mesothelioma. *Human Pathol* **18**: 544–557

Davis J (1979) The histopathology and ultrastructure of pleural mesotheliomas produced in the rat by injections of crocidolite asbestos. *Br J Exp Pathol* **60**: 642–652

Di Renzo MF, Narsimham RP, Olivero M, Bretti S, Giordano, S, Medico E, Gaglia P, Zara P and Comoglio PM (1991) Expression of the Met/HGF receptor in normal and neoplastic human tissues. *Oncogene* **6**: 1997–2003

Di Renzo MF, Poulsom R, Olivero M, Comoglio PM and Lemoine NR (1995) Expression of the *Met* hepatocyte growth factor receptor in human pancreatic cancer. *Cancer Res* **55**: 1129–1138

Dowrick PG, Prescott AR and Warn RM (1991) Scatter factor effects major changes in the cytoskeletal organization of epithelial cells. *Cytokine* **3**: 299–310

Duncan G, Riach RA, Williams MR, Webb SF, Dawson AP and Reddan JR (1996) Calcium mobilisation modulates growth of lens cells. *Cell Calcium* **19**: 83–89

Eagles G, Warn A, Ball RY, Baillie-Johnson H, Arakaki N, Daikuhara Y and Warn R (1996) Hepatocyte growth factor/scatter factor is present in most pleural effusion fluids from cancer patients. *Br J Cancer* **73**: 377–381

Fleury-Feith J. Kheuang L, Zeng L, Bignon J, Boutin C, Monnet I and Jaurand M-C (1995) Human malignant mesothelial cells: variability of ultrastructural features in established and nude mice transplanted cell lines. *J Pathol* **177**: 209–215

Fitzpatrick DR, Peroni DJ and Bielefeldt-Ohmann H (1995) The role of growth factors and cytokines in the tumorigenesis and immunobiology of malignant mesothelioma. *Am J Respir Cell Mol Biol* **12**: 455–460

Gak E, Taylor WG, Chan A and Rubin JS (1992) Processing of hepatocyte growth factor to the heterodimeric form is required for biological activity. *Fed Eur Biochem Soc Lett* **311**: 17–21

Galepp M, Christmas T, Mutsaers S, Manning L, Davis M and Robinson B (1993) Platelet-derived growth factor as an autocrine factor in murine malignant mesothelioma. *Eur Respir Rev* **3**: 192–194

Gerwin BI, Lechner JF, Reddel RR, Roberts AB, Robbins KC, Gabrielson EW and Harris CC (1987) Comparison of production of transforming growth factor-beta and platelet-derived growth factor by normal human mesothelial cells and mesothelioma cell lines. *Cancer Res* **47**: 6180–6184

Gherardi E, Sharpe M, Lane K, Sirulnik A and Stoker M (1993) Hepatocyte growth factor/scatter factor (HGF/SF), the *c-Met* receptor and the behaviour of epithelial cells. *Soc Exp Biol* **47**: 163–181

Han S, Stuart LA and Friezner Degen SJ (1991) Characterization of the DNF15S2 Locus on human chromosome 3: identification of a gene coding for four kringle domains with homology to hepatocyte growth factor. *Biochem* **30**: 9768–9780

Harvey P, Warn A, Newman P, Perry LJ, Ball RY and Warn RM (1996) Immunoreactivity for hepatocyte growth factor/scatter factor and its receptor, Met, in human lung carcinoma and malignant mesothelioma. *J Pathol* **180**: 389–394

Hembry RM, Murphy G, Cawston TE, Dingle JT and Reynolds JJ (1986) Characterization of a specific antiserum for mammalian collagenase from several species: immunolocalization of collagenase in rabbit chondrocytes and uterus. *J Cell Sci* **81**: 105–123

Hillerdal G (1983) Malignant mesothelioma 1982: review of 4710 published cases. *Br J Dis Chest* **77**: 321–343

Joseph A, Weiss GH, Jin L, Fuchs A, Chowdhury S, O'Shaugnessy P, Goldberg ID and Rosen EM (1995) Expression of scatter factor in human bladder carcinoma. *J Natl Cancer Inst* **87**: 372–377

Kolatsi-Joannou M, Woolf AS, Hardman P, White SJ, Gordge M and Henderson RM (1995) The hepatocyte growth factor/scatter factor (HGF/SF) receptor, *Met*

© Cancer Research Campaign 1998

transduces a morphogenetic signal in renal glomerular fibromuscular mesangial cells. *J Cell Sci* **108**: 3703–3714

Lee TC, Zhang Y, Aston C, Hintz R, Jagirdar J, Perle AM, Burt M and Rom WN (1993) Normal human mesothelial cells and mesothelioma cell lines express insulin-like growth factor I and associated molecules. *Cancer Res* **53**: 2858–2864

Miyazawa K, Tsubouchi H, Naka D, Takahashi K, Okigaki M, Arakaki N, Nakayama H, Hirono S, Sakiyama O, Takahashi K, Gohda E, Daikuhara Y and Kitamura N (1989) Molecular cloning and sequence analysis of cDNA for human hepatocyte growth factor. *Biochem Biophys Res Commun* **163**: 967–973

Miyazawa K, Shimomura T, Kitamura A, Kondo J, Morimoto Y and Kitamura N (1993) Molecular cloning and sequence analysis of the cDNA for a human serine protease responsible for activation of hepatocyte growth factor. *J Biol Chem* **268**: 10024–10028

Nakamura T, Nishizawa T, Hagiya M, Seki T, Shimonishi M, Sugimura A, Tashiro K and Shimizu S (1989) Molecular cloning and expression of human hepatocyte growth factor. *Nature* **342**: 440–443

Naldini L, Tamagnone L, Vigna E, Sachs M, Hartmann G, Birchmeier W, Daikuhara Y, Tsubouchi H, Blasi F and Comoglio PM (1992) Extracellular proteolytic cleavage by urokinase is required for activation of hepatocyte growth factor. *EMBO J* **11**: 4825–4833

Natali PG, Nicotra MR, Di Renzo MF, Prat M, Bigotti A, Cavalier R and Comoglio PM (1993) Expression of Met/HGF receptor in human melanocytic neoplasms: demonstrating the relationship to malignant melanoma tumour progression. *Br J Cancer* **68**: 746–750

Peto J, Hodgson J, Matthews F and Jones J (1995) Continuing increase in mesothelioma mortality in Britain. *Lancet* **345**: 535–539

Prat M, Narsimhan R, Crepaldi T, Nicotra M, Natali P and Comoglio P (1991) The receptor encoded by the human *c-Met* oncogene is expressed in hepatocytes, epithelial cells and solid tumours. *Int J Cancer* **49**: 323–328

Rong S, Bodescot M, Blair D, Dunn J, Nakamura T, Mizuno K, Park M, Chan A, Aaronson S and Vande Woude G (1992) Tumorigenicity of the Met proto-oncogene and the gene for hepatocyte growth factor. *Mol Cell Biol* **12**: 5152–5158

Rong S, Jeffers M, Resau JH, Tsarfaty I, Oskarsson M and Vande Woude GF (1993) Met expression and sarcoma tumorigenicity. *Cancer Res* **53**: 5355–5360

Rosen EM, Nigam SK and Goldberg ID (1994) Scatter factor and the *c-Met* receptor: a paradigm for mesenchymal/epithelial interaction. *J Cell Biol* **127**: 1783–1787

Sonnenberg E, Meyer D, Weidner KM and Birchmeier C (1993) Scatter factor/hepatocyte growth factor and its receptor, the c-Met tyrosine kinase, can mediate a signal exchange between mesenchyme and epithelia during mouse development. *J Cell Biol* **123**: 223–235

Stoker M and Perryman M (1985) An epithelial scatter factor released by embryo fibroblasts. *J Cell Sci* **77**: 209–223

Stoker M, Gherardi E, Perryman M and Gray J (1987) Scatter factor is a fibroblast-derived modulator of epithelial cell mobility. *Nature* **327**: 239–242

Tsarfaty I, Rong S, Resau JH, Rulong S, da Silva PP and Vande Woude G (1994) The Met proto-oncogene mesenchymal to epithelial cell conversion. *Science* **263**: 98–101

Tsubouchi H, Niitani Y, Hirono S, Nakayama H, Gohda E, Arakaki N, Sakiyama O, Takahashi K, Kimoto M, Kawakami S, Setoguchi M, Tachikawa T, Shi S, Arima T and Daikuhara Y (1991) Levels of the human hepatocyte growth factor in serum of patients with various liver diseases determined by an enzyme-linked immunosorbent assay. *Hepatology* **13**: 1–5

van Gelder T, Hoogsteden H and Vanderbroucke J (1991) The influence of the diagnosis technique on the histopathological diagnosis in malignant mesothelioma. *Virchows Arch A (Pathol Anat)* **418**: 315–317

Versnel MA, Hagemeijer A, Bouts MJ, van der Kwast TH and Hoogsteden HC (1988) Expression of c-sis PDGF B-chain and PDGF A-chain in ten human mesothelioma cell lines derived from primary and metastatic tumors. *Oncogene* **2**: 601–605

Webb CP, Lane K, Dawson AP, Vande Woude GF and Warn RM (1996) C-Met signalling in an HGF/SF-insensitive variant MDCK cell line with constitutive motile-invasive behaviour. *J Cell Sci* **109**: 2371–2381

Whitaker D, Papadimitriou JM and Walters MN (1982) The mesothelium and its reactions. A review. *CRC Crit Rev Toxicol* **10**: 81–144

Woolf AS, Kolatsi-Joannou M, Hardman P, Andermarcher E, Moorby C, Fine LG, Jat PS, Noble MD and Gherardi E (1995) Roles of hepatocyte growth factor/scatter factor and the Met receptor in the early development of the metanephros. *J Cell Biol* **128**: 171–184

Yamashita J-I, Ogawa M, Yamashita S-I, Nomura K, Kuramoto M, Saishoji T and Shin S (1994) Immunoreactive hepatocyte growth factor is a strong and independent predictor of recurrence and survival in human breast cancer. *Cancer Res* **54**: 1630–1633

Yoshimura T, Yuhki N, Wong MH, Skeel A and Leonard E (1993) Cloning, sequencing and expression of human macrophage stimulating protein, (MSP, MST 1), confirms MSP as a member of the family of kringle proteins and locates the MSP gene on chromosome 3. *J Biol Chem* **268**: 15461–15468

Zeng L, Fleury-Feith J, Monnet I, Boutin C, Bignon J and Jaurand MC (1994) Immunocytochemical characterisation of cell lines from human malignant mesothelioma. *Human Pathol* **25**: 227–234

© Cancer Research Campaign 1998